# Electronic Discharge Communication Tools Used in Pediatric Emergency Departments: Systematic Review

**DOI:** 10.2196/36878

**Published:** 2022-06-24

**Authors:** Lori Wozney, Janet Curran, Patrick Archambault, Christine Cassidy, Mona Jabbour, Rebecca Mackay, Amanda Newton, Amy C Plint, Mari Somerville

**Affiliations:** 1 Mental Health and Addictions Nova Scotia Health Dartmouth, NS Canada; 2 IWK Health Strengthening Transitions in Care Lab Halifax, NS Canada; 3 School of Nursing Dalhousie University Halifax, NS Canada; 4 Département de médecine d'urgence Centre intégré de santé et de services sociaux de Chaudière-Appalaches Levis, QC Canada; 5 Department of Pediatrics University of Ottawa Ottawa, ON Canada; 6 Children's Hospital of Eastern Ontario Ottawa, ON Canada; 7 Department of Pediatrics, Faculty of Medicine & Dentistry University of Alberta Edmonton, AB Canada; 8 Department of Pediatrics and Emergency Medicine University of Ottawa Ottawa, ON Canada

**Keywords:** emergency department, medical informatics, pediatric, systematic review, patient discharge summaries, patient-centered care, technology, hospital

## Abstract

**Background:**

Electronic discharge communication tools (EDCTs) are increasingly common in pediatric emergency departments (EDs). These tools have been shown to improve patient-centered communication, support postdischarge care at home, and reduce unnecessary return visits to the ED.

**Objective:**

This study aimed to map and assess the evidence base for EDCTs used in pediatric EDs according to their functionalities, intended purpose, implementation context features, and outcomes.

**Methods:**

A systematic review was conducted following PRISMA (Preferred Reporting Items for Systematic Reviews and Meta-Analyses) procedures for identification, screening, and eligibility. A total of 7 databases (EBSCO, MEDLINE, CINAHL, PsycINFO, EMBASE Scopus, and Web of Science) were searched for studies published between 1989 and 2021. Studies evaluating discharge communication–related outcomes using electronic tools (eg, text messages, videos, and kiosks) in pediatric EDs were included. In all, 2 researchers independently assessed the eligibility. Extracted data related to study identification, methodology, settings and demographics, intervention features, outcome implementation features, and practice, policy, and research implications. The Mixed Method Appraisal Tool was used to assess methodological quality. The synthesis of results involved structured tabulation, vote counting, recoding into common metrics, inductive thematic analysis, descriptive statistics, and heat mapping.

**Results:**

In total, 231 full-text articles and abstracts were screened for review inclusion with 49 reports (representing 55 unique tools) included. In all, 70% (26/37) of the studies met at least three of five Mixed Method Appraisal Tool criteria. The most common EDCTs were videos, text messages, kiosks, and phone calls. The time required to use the tools ranged from 120 seconds to 80 minutes. The EDCTs were evaluated for numerous presenting conditions (eg, asthma, fracture, head injury, fever, and otitis media) that required a range of at-home care needs after the ED visit. The most frequently measured outcomes were knowledge acquisition, caregiver and patient beliefs and attitudes, and health service use. Unvalidated self-report measures were typically used for measurement. Health care provider satisfaction or system-level impacts were infrequently measured in studies. The directionality of primary outcomes pointed to positive effects for the primary measure (44/55, 80%) or no significant difference (10/55, 18%). Only one study reported negative findings, with an increase in return visits to the ED after receiving the intervention compared with the control group.

**Conclusions:**

This review is the first to map the broad literature of EDCTs used in pediatric EDs. The findings suggest a promising evidence base, demonstrating that EDCTs have been successfully integrated across clinical contexts and deployed via diverse technological modalities. Although caregiver and patient satisfaction with EDCTs is high, future research should use robust trials using consistent measures of communication quality, clinician experience, cost-effectiveness, and health service use to accumulate evidence regarding these outcomes.

**Trial Registration:**

PROSPERO CRD42020157500; https://www.crd.york.ac.uk/prospero/display_record.php?RecordID=157500

## Introduction

### Communication Is the Cornerstone of Care

Pediatric patients account for a significant proportion of all emergency department (ED) visits (30% in Canada, 31.2% in Korea, and 20.5% in the United States) worldwide [1-3]. Previous studies and reports have reported that 58% [4] to 87% [5] of all pediatric patients visited the ED with nonurgent conditions, meaning that most are discharged home, where parents are expected to manage care. As a result, the discussions that ED staff have with patients and caregivers during the discharge process regarding what care is required after the ED visit is a significant component of safe practice and quality patient care [6].

Discharge communication among providers, parents, and patients occurs at multiple points during an ED visit, and sharing information related to diagnosis, prognosis, treatment plans, and anticipated course of illness is critical for successful discharge to home [7]. Poor compliance and lack of comprehension of discharge instructions have significant clinical implications, including unfinished treatment, poor pain management, and possible progression of illness [8]. Deficits in the understanding of discharge instructions are widely documented, ranging from 24% of discharged patients with poor understanding of their follow-up plan [9] to patients correctly identifying only 59% of instructions [10]. A review of 48 pediatric ED studies determined that one-third to almost half of the parents who had visited the ED with their child made medication dosing errors during post-ED home care [11]. Within the complex, fast-paced, highly stressful, and highly distracting ED environment, discharge communication can take place in as little as 76 seconds [12]. As a result, interventions to improve communication must balance reliability with flexibility across a wide range of clinical presentations.

### Using Technology to Improve Discharge Communication and Outcomes

The use of information and communication technologies (ICTs) in pediatric health care institutions is increasing, as are the multiple ways in which different technologies are deployed. Examples include computer kiosks, mobile apps, interactive television and whiteboards, electronic health records, videos, websites, and automated email [13,14]. Technologies create new opportunities for communication and dynamic updates for patient care; however, at the same time, they can also introduce potential interruptions or changes in clinical workflow [15]. Greater emphasis on the interplay between the social (people, values, and norms), technical (tools, hardware, equipment, and processes), and behavioral (routines, roles, and tasks) aspects of ICT implementation in discharge communication could help address some of these barriers [16].

To improve the experience of care during and after an ED visit, there is a great need to better leverage the strengths of technologies to support efficient discharge processes, particularly for nonurgent visits. However, few guidelines exist to support health care institutions in decision-making and implementation planning for such technologies. Research on the use of ICT to support care transitions is predicted to grow rapidly as patients and clients increasingly demonstrate preferences for the use of these technologies in their care [17]. Health care providers also recommend better and more appropriate use of ICT to support families in self-managing care at home [18]. Despite the communication challenges faced by families during this transition point, strain on existing ED resources and the lack of standards and implementation guidelines remain significant barriers to the widespread adoption of electronic discharge communication tools (EDCTs) in pediatric emergency contexts. Systematic reviews of traditional pediatric discharge communication practices [19,20] and computer technology have enabled discharge communication outside the ED [21]; however, to our knowledge, there has not been a comprehensive review of how EDCTs are being used to support and guide pediatric emergency discharge communication.

### Objectives and Research Questions

This systematic review of academic literature was undertaken to identify, appraise, and describe the use of EDCTs in pediatric emergency contexts. Our goal is to advance the knowledge base for researchers, technology designers, and decision makers to anticipate the impact of their communication tools on the clinical workflow and the optimal ways to measure impact (Textbox 1).

**Guiding questions for review.**

**Guiding questions**

- What electronic discharge communication tools (EDCTs) have been evaluated in pediatric emergency departments and published following peer review?
- What are the features and technical components of these EDCTs?
- What outcome measures are being examined in the EDCT literature?
- What is the methodological quality of the studies conducted on EDCTs?
- What are the implementation context features where EDCTs have been tested?
- What are the priority research, practice, and policy actions advocated by the authors of research in this domain?

## Methods

### Approach

The PRISMA (Preferred Reporting Items for Systematic Reviews and Meta-Analyses) [22] guidelines were followed, and the review was registered with PROSPERO CRD42020157500.

### Data Sources and Search Strategy

A comprehensive search strategy using the Population Intervention Comparator Outcome framework [23] was codeveloped with an experienced information technician. The search terms were intentionally broad to capture the range of EDCTs. Namely, terms included technology (eg, electronic documents or web-forms, mobile device apps, patient portals, notification systems, text messages or SMS notifications, interactive online decision trees, automated email, and video-based programs) used to prompt communication between caregivers/patients and ED staff about the ED visit, and structure the exchange of information, or promote compliance, education, and information sharing about what care should be given after the ED visit is over. A total of 7 databases of publisher-controlled and gray literature were searched: EBSCO, MEDLINE, CINAHL, PsycINFO, EMBASE, Scopus, and Web of Science. The original search was conducted in June 2019 and was updated in August 2021 to capture current evidence. Records from 1989 onward were included. Multimedia Appendix 1 presents the sample search strategy. The reference lists of systematic reviews were also hand searched for primary studies.

### Eligibility Criteria

We used a broad definition of EDCTs, including tools that prompt communication between caregivers and patients and ED staff about the ED visit and structure the exchange of information and promote compliance, education, and information sharing about what care should be given after the ED visit is over. We did not limit the search to a particular technology modality; therefore, tools including web-based documents or web-forms, mobile device apps, patient portals, notification systems, text messages or SMS notifications, interactive web-based decision trees, automated email, and video were eligible for inclusion. As telephone-based services are part of Health Canada’s definition of eHealth, we included phone-based services under the broad umbrella of *electronic tools*.

Specific inclusion and exclusion criteria are presented in Textbox 2.

**Inclusion and exclusion criteria.**

**Inclusion criteria**

- Electronic discharge communication tools (EDCTs) designed for use during or after an emergency department (ED) visit
- Studies or abstracts that reported outcome data on at least one communication process or communication outcome targeted by the EDCT
- Studies conducted in pediatric ED
- Studies conducted in mixed EDs (adult and pediatric) as long as the EDCT was evaluated in a pediatric population, and outcomes were disaggregated for analysis
- Publicly available in English

**Exclusion criteria**

- Educational intervention given to the patient or caregiver while in the ED but not directly associated with the patient’s illness presentation (ie, seatbelt safety)
- Tools only targeting health care provider to health care provider communication
- Reviews, meta-analyses, research protocols, editorials, and case-studies

### Screening

Eligibility screening was performed using Covidence software [24]. All titles and abstracts were independently reviewed by 2 reviewers. Discrepancies regarding which studies to include in full-text reviews were resolved by discussion. A total of 2 reviewers independently assessed the full texts for inclusion. Discrepant classifications were resolved through discussion.

### Data Abstraction and Analysis

The team co-designed and piloted a structured data extraction table with the 4 studies included in the review. The form included sections on (1) *study identification* (eg, type of publication, year, and author); (2) *methods* (eg, study design and sample size); (3) *delivery settings and demographics* (eg, ED features, age, setting characteristics, and computer proficiency); (4) *intervention design* (eg, design framework, frequency and duration of interaction, tailoring, bidirectional functionality, content, tool, and primary technology modality); (5) *outcomes* (eg, category of outcome measure, follow-up schedule, and covariates); (6) *implementation* (eg, who administered the tool, training requirements, interoperability, and cost); and (7) *practice, policy, and research implications* extracted verbatim from the Discussion and Conclusions sections.

As a broad range of study designs was anticipated, the Mixed Method Appraisal Tool (MMAT) version 2018 [25] was used for methodological quality appraisal. The MMAT is a 21-item checklist with 5 research designs. Each research design category has 5 quality criteria that are appraised as yes (criterion met) and no (criterion not met or cannot tell [unable to tell from text if the criterion was met or not]). Assigning studies an overall numerical score based on the ratings of each criterion is discouraged, because a single number cannot provide insight into which aspects of the study methodology are problematic [26]. Instead, we classified studies as having lower methodological quality when they met ≤60% of the MMAT criteria and higher quality when they met >60% of the criteria. This is consistent with the approaches outlined by the MMAT authors [26].

A reviewer independently conducted data extraction and MMAT scoring for all full-text articles. As a quality assurance measure and to ensure the accuracy of extraction, a second reviewer independently extracted data from a randomly selected subset of 30% of full texts. The results were compared, disagreements were resolved by discussion, and additional instructions for the coder were updated.

Following standard practices for systematic reviews–included [27] studies were synthesized using several approaches: (1) structured tabulation to explore patterns in the raw data, (2) vote counting of raw data (eg, reporting on the frequency of different study features), (3) constructing a common rubric to transform qualitative data (eg, lengthy descriptions of the technology features) into a simplified quantitative form (eg, assigning tools to a modality category), (4) descriptive statistics (eg, range, mean, or median) to summarize quantitative data, (5) inductive thematic analysis (eg, hierarchical coding of verbatim policy, practice, and research implications), and (6) visual depiction of summary data.

## Results

### Overview

**Figure 1:**
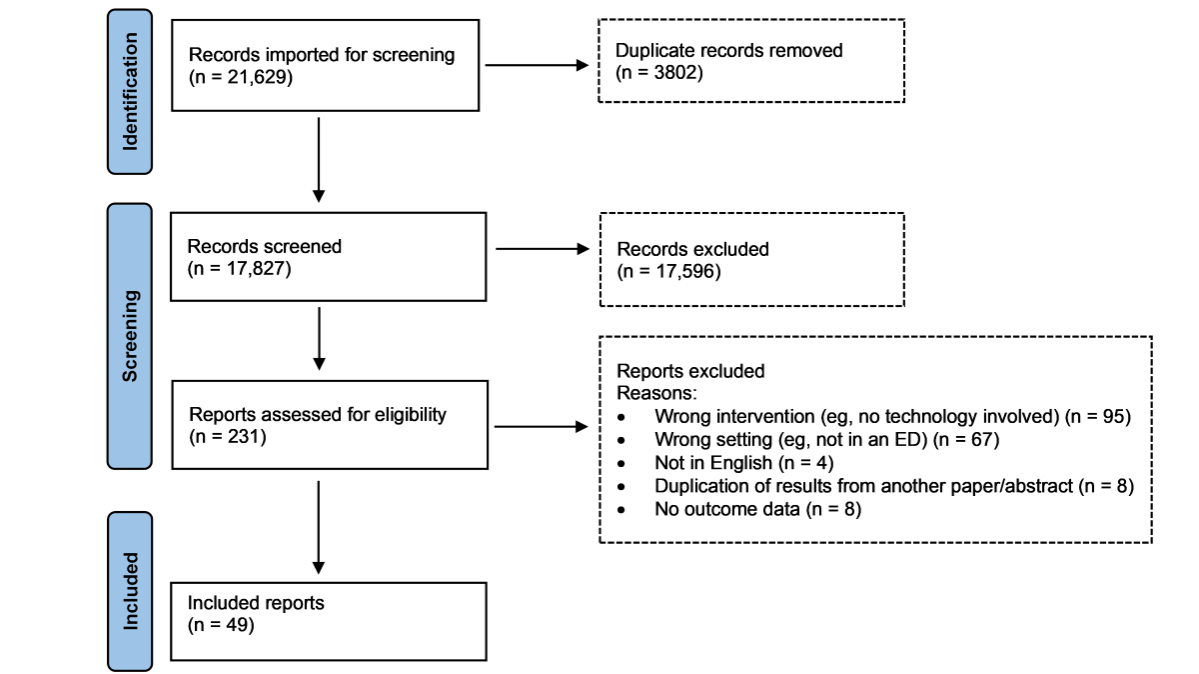
PRISMA (Preferred Reporting Items for Systematic Reviews and Meta-Analyses) flow diagram for the systematic review detailing the database searches, the number of abstracts screened, and the full texts retrieved. ED: emergency department.

Duplicates were excluded, and 17,827 potential reports were returned. Hand searching of the reference lists of 15 related systematic reviews produced no additional eligible full-text reports. A total of 231 reports were read in full, with 182 (78.8%) excluded, leaving 49 (21.2%) reports detailing findings for 55 unique EDCTs. A flowchart of the process is shown in Figure 1.

### Study Characteristics

The studies were conducted in 8 countries between 1989 and 2021 (Table 1). The intervention group sample size ranged from 3 to 4091 participants or events (median 95). In all, 62% (34/55) of the studies were conducted in the United States and 20% (11/55) in Canada, with the remainder (10/55, 18%) conducted in Australia, China, the Netherlands, South Korea, and the United Kingdom. A study did not report the country of origin. Interventions were evaluated using randomized controlled trial designs in 58% (32/55) of the cases, nonrandomized trials and cohort designs in 22% (12/55), quantitative descriptive studies in 20% (10/55), and mixed methods at an instance 2% (1/55).

**Table 1:** Study characteristics and key features of the interventions.

Module and author and year		Country		Condition		Sample size, N^a^		ED^b^		Purpose		Focus		Main outcomes		Timing		Frequency		Duration	
**Kiosk**																					
	Fine et al, 2009 [28]	United States		Otitis media, urinary tract infection, head trauma, and asthma		1072		Mixed		Empower patients to electronically provide historical aspects of a child’s illness and adhere to evidence-based care		Produced summary forms for parent-provided historical data, suggestions about how to communicate proactively with staff, summary of the child’s symptoms, medications, and allergies and listed a tailored action plan		During ParentLink use, documentation of pain significantly improved (28% incomplete [control] vs 15% [intervention]; *P*=.003)		During		NR^c^		10 minutes	
	Joshi et al, 2009 [29]	United States		Asthma		99		Pediatric		Teach children about asthma and its management		General education		Tool was effective in improving the asthma knowledge of young patients and those having lower baseline knowledge		During		NR		NR	
	Kearns et al, 2021 [30]	United States		Asthma		4191		Mixed		To determine the impact of an electronic intervention on asthma care quality		Measured patients’ severity level and provided most appropriate care pathway based on severity score and provided prompts for medication		Cumulative use was associated with significantly reduced odds of hospital admission		During		Once		NR	
	Kwok- et al, 2018 [31]	United States		Asthma		31		Pediatric		To (1) capture from caregivers the critical information necessary to categorize the child’s asthma severity, (2) deliver asthma education to families, and (3) generate guideline-based chronic asthma management plans for the caregivers and ED physicians		General education		Long-term controller medications prescribing and screening provision for 19 of 31 (61%) and 17 of 31 (55%) patients, respectively		During		Once		7 minutes	
	Morrison et al, 2021 [32]	United States		Asthma		3084		Mixed		To increase the number of families receiving asthma education and impact on workflow		General education (signs and symptoms)		Increase in number of families receiving education and trending decrease in ED visits		During		NR		NR	
	Mortenson et al, 2016 [33]	Canada		Mind-brain injury		38		Pediatric		To reduce parental reports of postconcussion symptoms and caregiver anxiety and stress		Service recommendations linked to e-mental health care based on needs. The resources were customized by patient age, sex, language, and region.		No significant difference between the groups at 3 months after injury in postconcussion symptoms and family stress		After		NR		NR	
	Polihronis et al, 2016 [34]	Canada		Mental health		500		Pediatric		Patient’s perceived feasibility of using web-based screening tool to tailor discharge recommendations; newly developed web-based HEADS-ED^d^ screening tool in the ED		Unclear		No significant differences in HEADS-ED scores were found between participants in phases 1 and 2		During		Once		NR	
	Porter et al, 2004 [35]	United States		Asthma		65		Pediatric		Designed a patient-centered interface to allow parents of children with asthma to be active providers of knowledge and promoters of quality of care in the ED and improve quality of care		Summarizes parent-provided historical data, likely ED-based actions and suggestions for the parent on proactive communication with ED providers. Creates a provider-centric form summarizing symptoms, medications, and allergies of the child and listing a tailored plan for evaluation and treatment on a single diagnostic category.		The tool successfully links patent’s data to guideline recommendations and identifies data critical to health improvements		After		NR		12 minutes	
	Porter et al, 2008 [36]	United States		Head trauma; dysuria; ear pain; respiratory symptoms and history of asthma; fever		654		Pediatric		To determine impact of intervention on error rate of ordering and prescribing medication		Parent enters information and is given a tailored summary form with all relevant history, suggestions for proactive communication, and a tailored list of suggestions for the provider to review.		No significant difference between those using the tool and the control group		During		Once		NR	
	Sinha et al, 2014 [37]	United States		Nonspecific		200		Pediatric		To determine if a triage kiosk was more efficient than standard nurse-initiated triage and to compare accuracy of medical history and patient satisfaction		Triage questions supplemented by audio prompts in the patient’s language of choice.		The mean (SD) time to enter medical history data by the kiosk group was significantly shorter than the standard nurse triage group (94.38, SD 38.61 vs 126.72, SD 62.61 seconds; *P*=.001)		During		Once		2 minutes	
	Porter et al, 2006 [38]	United States		Nonspecific		131		Pediatric		To determine the effect of ParentLink parent satisfaction with care experience related to communication with providers and adoption of guideline-endorsed process of care		Parents report symptoms, medications, and unmet needs.		No significant differences in partnership problems (ie, provider and caregiver communication)		After		Once		NR	
**Video**																					
	Baker et al, 2009 [39]	United States		Fever		140		Pediatric		Improve knowledge and ability to home-manage fever and reduce medically unnecessary return ED visits for febrile episodes		Methods for taking a temperature, outlines indications for contacting a physician, refutes common parental misconceptions about fever, and identifies methods to comfort a febrile child.		The fever video had a significant improvement in several measures relating to knowledge and attitudes about childhood fever		During		Once		11 minutes	
	Belisle et al, 2019 [40]	Canada		Otitis media		77		Mixed		To determine if video discharge instructions were associated with improved symptomatology, functional outcome, and knowledge compared with a paper handout		Instructions on management of pain and fever		Median symptom severity score in the video group was significantly lower than the paper group, even after adjusting for preintervention AOM-SOS and medication (analgesics and antibiotics) given by caregivers 8 (7-13) vs 10 (7-13), respectively, *P*=.004		During		NR		NR	
	Bloch and Bloch, 2013 [41]	United States		Fever		107		Pediatric		Improve caregiver’s comprehension of their child’s medical condition, treatment, and follow-up and improve caregiver satisfaction		General education (eg, symptoms and treatment options)		The group receiving video instructions scored significantly higher in the ED immediately following intervention (12.2 vs 8.9) and 2 to 5 days after discharge (11.1 vs 7.8)		During		NR		3 minutes	
	Bloch and Bloch, 2013 [41]	United States		Vomiting or diarrhea		68		Pediatric		Improve caregiver’s comprehension of their child’s medical condition, treatment, and follow-up and improve caregiver satisfaction		General education (eg, symptoms and treatment options)		Intervention group scored significantly higher on knowledge (12.2 vs 8.9) and 2 to 5 days after discharge (11.1 vs 7.8)		During		NR		3 minutes	
	Bloch and Bloch, 2013 [41]	United States		Asthma		41		Pediatric		Improve caregiver’s comprehension of their child’s medical condition, treatment, and follow-up and improve caregiver satisfaction		General education (eg, symptoms and treatment options)		Intervention group video scored significantly higher on knowledge (12.2 vs 8.9) and 2 to 5 days after discharge (11.1 vs 7.8). At follow-up, 29% of the written and 42% of the video groups rated their discharge instructions as being extremely helpful. I		During		NR		3 minutes	
	Boychuk et al, 2006 [42]	United States		Asthma		590		Mixed		Teach and reinforce basic self-management concepts		Covers signs and symptoms of asthma, pathophysiology, treatment (including medications), how to use the asthma action plan, and demonstration of equipment use.		Number of patients possessing a written asthma action plan increased from 48 to 322		During		NR		6 minutes	
	Golden-Plotnik et al, 2018 [43]	Canada		Fracture		117		Pediatric		To determine whether an educational video was superior to standard care for pain management		Recognition of pain, over-the-counter analgesic dosing and indications, risks and safety in children, and signs and symptoms of pain and misconceptions about treating pain in children		The educational video change in knowledge (delta)=2.3 (95% CI 1.3-3.3); *P*<.001		After		NR		Unlimited for 120 hours	
	Hoek et al, 2020 [44]	Netherlands		Nonspecific		174		Mixed		Determine whether written and video instructions improve recall on how to use analgesics		Link to web-based video with information on analgesics dosing and scheduling aimed to refute prejudice about use		Significant difference in written over oral but video was only viewed by 5% of participants		After		Unlimited		NR	
	Ismail et al, 2016 [45]	United States		Fever; head injury		31		NR		Improve caregiver’s comprehension of their child’s diagnosis, treatment, and follow-up care		Information about diagnosis, treatment, disease process, and discharge instruction.		The intervention group had a significantly higher percentage of correct answers on postintervention tests (median 99.89) than the control (median 75.73) *P*<.001		During		Once		6 minutes	
	Jové-Blanco et al, 2021 [46]	Spain		Gastroenteritis		69		Mixed		To evaluate if the video improved comprehension; patients were satisfied and decreased return visits		General education (eg, etiology, treatment, signs and symptoms, after-care, and reasons to reconsult)		Greater improvement in knowledge among intervention group		During		Once		2 minutes	
	Jung et al, 2011 [47]	South Korea		Head injury		95		Pediatric		Improve discharge instruction comprehension		General education		Video explanation to parents with children with minor head trauma in the pediatric EDs can increase the satisfaction compared with previous paper-using instruction method		During		Once		NR	
	Ladde et al, 2013 [48]	United States		Asthma		29		Pediatric		To determine whether an educational video compared with standard reading materials would better educate pediatric asthma patient’s primary caregivers and if this would affect 30-day ED revisits		General information		Admit rate for visit was 24.1% (26.7% video vs 21.4% paper), *P*=.74		During		NR		NR	
	Lawrence et al, 2009 [49]	United States		Nonspecific		587		Pediatric		To decrease the number of medically unnecessary return visits to the pediatric ED		Reminder to take medication		Of all return visits to the pediatric ED within 72 hours of discharge, 13% were deemed unnecessary for patients receiving handwritten instructions compared with 15% for patients receiving computer-generated instructions (*P*=.50)		After discharge		Daily		NR	
	Lion et al, 2015 [50]	United States		Nonspecific		142		Mixed		To determine the effect of video interpretation on comprehension, parent-reported quality of communication, and frequency of use of professional translators		Unclear		Those in the video arm were more likely to name the child’s diagnosis correctly than those in the telephone arm (85/114, 74.6% vs 52/87 59.8%; *P*=.03) and less likely to report frequent lapses in interpreter use (2/117, 1.7% vs 7/91, 7.7%; *P*=.04)		During		NR		NR	
	Macy et al, 2011 [51]	United States		Asthma		53		Pediatric		To increase asthma knowledge, parental sense of asthma control, parental report of asthma symptoms, and decrease health care use		Unclear		Improvement in asthma knowledge at follow-up was realized for low-literacy parents regardless of the type of educational intervention (*P*<.001)		During		Once		20 minutes	
	Mian et al, 2016 [52]	United Kingdom		Oncology		32		Mixed		To decrease the time to recognize fever-neutropenia to reduce ed visits		Discussion and recommendation for symptom management and activity participation. Families provided with additional web links and education		Education of the patient’s caregiver improved their understanding by 84% and significantly decreased their time for symptom recognition and ED presentation		During		Once		8 minutes	
	Stevens et al, 2012 [53]	United States		Pain		59		Pediatric		To evaluate the effectiveness of a 6-minute instructional video for parents that targets common misconceptions about home pain management		General education		Significantly more parents provided at least one dose of pain medication to their children after watching the educational video: 96% vs 80% (difference 16%, 95% CI 7.8%-31.3%)		During		NR		NR	
	Wood et al, 2017 [54]	United States		Gastroenteritis; bronchiolitis; fever		41		Pediatric		To determine if the intervention improved knowledge about diagnosis, treatment, illness duration, and when to seek further medical care		The videos described symptoms associated with the diagnosis, treatment of the symptoms expected illness duration, and when to seek further medical care.		Both groups showed improvement but video group had statistically more recall		During		Once		3 to 5 minutes	
	Wood et al, 2020 [55]	United States		Fever; gastroenteritis; bronchiolitis		75		Pediatric		To determine if adding a video component to standard care improved knowledge acquisition		Information on child’s diagnosis, treatment illness duration, and when to seek further care		Video group achieved significantly higher scores on the posttest survey than the standard care group, particularly regarding treatment and when to seek further medical care		During		Once		5 minutes	
	Zorc et al, 2009 [56]	United States		Asthma		217		Pediatric		To determine if the intervention would address beliefs and barriers to follow-up asthma care among inner-city families		General education on What is asthma? How can asthma be controlled? What are the benefits of controlling asthma?		Intervention participants were more likely to endorse beliefs about the benefits of follow-up than controls		During		Once		12 minutes	
**Phone**																					
	Bucaro and Black, 2014 [57]	United States		Nonspecific		630		Pediatric		Increase parental understanding of ED discharge instructions so that parents can successfully and safely manage their child’s care at home		General education (eg, symptoms and treatment options)		In all, 93% of parents found that after the follow-up call, they had an improved understanding of their child’s illness or injury		After		Once		NR	
	Chande and Exum, 1994 [58]	United States		Pneumonia; croup, asthma; bronchiolitis; vomiting; fever		133		Pediatric		Improve parental compliance with primary care follow-up		Reminders to fill their prescriptions, to call regular physicians, and to follow any other instructions documented on the discharge sheet		No significant difference between groups on frequency of filling prescriptions		After		Once		NR	
	Goldman et al, 2014 [59]	Canada		Nonspecific		171		Pediatric		To examine whether a follow-up telephone call by a non–health care provider from the ED within 24 hours after a child’s discharge can reduce the rate of returning to the ED within 72 hours		Information about the child’s medical condition after discharge and community follow-up and responding to parents’ questions		The outcome measure was found to be in contrary to our hypothesis. We found return visits to the ED in 24 (14%) of the children in the study group compared with only 14 (7%) in the control group (*P*<.03)		After		Up to 10 trials in difference hours		NR	
	Jones et al, 1989 [60]	United States		Otitis media		14		Pediatric		To evaluate 2 clinical nursing interventions designed to increase compliance with follow-up care referrals for patients		Health Belief Model phone intervention		Participants who received the intervention were much more likely than control participants to comply with a follow-up referral appointment		During		Once		NR	
	Jones et al, 1989 [60]	United States		Otitis media		12		NR		To evaluate 2 clinical nursing intervention designed to increase compliance with follow-up care referrals for patients		Health Belief Model phone intervention		Participants who received the intervention were much more likely than control participants to comply with a follow-up referral appointment		After		Once		NR	
	Khan et al, 2004 [61]	Australia		Asthma		136		Pediatric		To improve asthma management and control		Asthma severity information. Educational topics on self-management. Collected information about barriers to optimal care and engaged ED staff in selecting recommended preventive medications with an option to print		Intervention group children were significantly more likely than controls to possess (87.5% vs 72.3%; *P*=.002) a written action plan		After		Once		NR	
	Wong et al, 2004 [62]	China		Fever, respiratory, or gastrointestinal condition		395		Pediatric		To determine if ED nurse follow-up (via phone call) helped to change health outcome and health care use		Assessment of symptoms and decision on management options.		Significantly different between intervention and control groups on improvement of the condition and ED visit within 30 days		After		Twice		NR	
**Web-based**																					
	Babcock et al, 2017 [63]	United States		Mild traumatic brain injury		13		Pediatric		Promote concussion recovery for adolescents through education and training in self-management and effective coping		Symptom and activity monitoring to promote self-management. Educational modules that provided anticipatory guidance and techniques to effectively manage these consequences using cognitive reframing, relaxation training, and problem solving.		Significant improvement in symptoms over the 4-week program (adolescent: *P*<.001; parent *P*=.004)		After		Unlimited		NR	
	Goldman et al, 2005 [64]	Canada		Nonspecific		303		Pediatric		To determine whether the internet could be used to report information on bacterial cultures taken in the pediatric ED and whether parents would use the tool to gain access to personalized culture results		Access to the participant’s culture results using a unique ID and password		186 (61%) parents accessed the internet-system after mean 94 hours (range 1 minute-611 hours) after posting		After		NR		NR	
	Hart et al, 2019 [65]	Canada		Fever		77		Pediatric		To determine if web-based interventions improve recognition and management of fever at home, leading to decreased parental anxiety and possibly fewer unnecessary ED visits by measuring knowledge acquisition and satisfaction		Computer-automated feedback regarding childhood fever		Mean pretest to immediate posttest gain score of 3.5 (SD 4.1); *P*<.001		During		NR		NR	
**Computer-based**																					
	Alqudah, 2014 [66]	Australia		Fever		95		Mixed		Evaluate the impact of a health literacy–modified fever education program on parents or carers’ fever knowledge, anticipated fever management practices, and ED or primary care presentations		Pharmacological and nonpharmacological fever management practices, the correct way to measure a child’s body temperature, and general knowledge about fever		No statistically significant difference		During		NR		NR	
	Alqudah, 2014 [66]	Australia		Fever		3		Mixed		Evaluate the impact of a health literacy–modified fever education program on parents or carers’ fever knowledge, anticipated fever management practices, and ED or primary care presentations		Pharmacological and nonpharmacological fever management practices, the correct way to measure a child’s body temperature, and general knowledge about fever		No statistically significant difference		During		NR		NR	
	Fernandez et al, 2011 [67]	United States		Asthma		27		Pediatric		Improve effectiveness and retention of asthma education for children		General education		Factors motivating participation included the need to be in the ED, parental involvement in the process, and effective use of technology. Barriers identified were fatigue of child, unavailability of parent, and ED visit during uncovered educator hours		After		As many times as they liked		NR	
	Golden-Plotnik et al, 2018 [43]	Canada		Fracture		111		Pediatric		To determine whether a web-based module was superior to standard care for pain management at home		General education		The web-based module group showed change in knowledge (delta)=1.6 (95% CI 0.5-2.6); *P*=.002		After		NR		Unlimited for 120 hours	
	Hart et al, 2019 [65]	Canada		Fever		79		Pediatric		To determine if web-based interventions improve recognition and management of fever at home, leading to decreased parental anxiety and possibly fewer unnecessary ED visits		Computer-automated feedback regarding childhood fever (noninteractive)		Mean pretest to immediate posttest gain score of 3.5 (4.2); *P*<.001		During		NR		NR	
**Text message or SMS**																					
	Sockrider et al, 2006 [68]	United States		Asthma		263		NR		To determine if the intervention group would have greater confidence to manage asthma, better primary care follow-up, and fewer return ED visits		The intervention includes universal and tailored content, and the educator has the flexibility to navigate the content based on the individual child or family’s needs and questions		The confidence level to prevent asthma episodes and keep them from getting worse was significantly higher in the intervention group at 14 days after intervention		During		Once		NR	
	Boyd et al, 2013 [69]	United Kingdom		Fracture		25		NR		To investigate whether text message reminders improve pain management in children after discharge from the ED		Reminders to improve pain management		The mean number of analgesia doses administered to the text message group was 7.6 vs 4.9 in the control group, *P≤*.05		After		Twice		NR	
	Lee et al, 2011 [70]	United States		Asthma		7		Mixed		To demonstrate that text message medication reminders will improve medication adherence		General discharge information		Results did not demonstrate a significant difference of means (paired 2-tailed *t* test) between pre- and post–text messaging reminders		After		Multiple		NR	
	Malbon et al, 2013 [71]	United States		Nonspecific		2440		Pediatric		Encouraging primary care follow-up at an adolescent health center for adolescents who sought care at an ED		Reminder		Text messaging is a feasible and effective tool for increasing outpatient follow-up after an ED visit at a primary care facility, potentially relieving an additional burden on the ED and promoting health care in the transition to adult medicine		After		Multiple		NR	
	Salinero, 2012 [72]	United States		Nonspecific		61		Pediatric		To evaluate whether a text message reminder to the caregivers after discharge from the pediatric ED improved compliance with recommended primary care follow-up		Reminder to follow-up with their primary care physician		There was no significant difference in follow-up in the standard treatment group 19/62 (31%) vs the text message intervention group 16/61 (26%); *P*=.69		After		Once		NR	
	Wolff et al, 2016 [73]	United States		Pelvic inflammatory disease		47		Mixed		To test the effect of text message reminders on adolescent patients’ adherence to the recommended post-ED follow-up care		Personalized reminders to schedule and attend a follow-up appointment.		Patients receiving text message reminders were more likely to follow up compared with the standard group (relative risk=2.9, 95% CI 1.4-5.7)		After		4 times		NR	
**Game-based**																					
	Taylor et al, 2015 [74]	Canada		Nonspecific		533		Pediatric		To determine level of patient satisfaction and improvement in pain management and treatment while in the ED		Patients and parents view videos selected by the triage nurse in response to perceived patient need. The videos reframe and demystify injury and illness, inform about medical procedures and processes, and introduce important coping skills. Permits individual messaging to both parents and patients via iPads.		Intervention participants showed significant improvements in pain control and both patient and parent satisfaction		During		Once		NR	
**Mobile app**																					
	Farooqui et al, 2017 [75]	NR		Asthma		98		NR		Effect of reminders on health care use		Reminders for medication and electronic treatment plan		Reported improvement in asthma management was greater in AsthmaCare participants (79% vs 62%; *P*=.06), along with greater daily use of treatment plans (29% vs. 11%; *P*=.01)		After		NR		NR	
**Photo documentation**																					
	Lund et al, 2013 [76]		Canada		Skin infection		244		Pediatric		To determine whether photo documentation improves the duration of outpatient treatment		Educational messages on basic facts about asthma, roles of medications, and patient skills.		No differences in the rate for completion and therapeutic failure were observed (71% vs 68% and <1% for both, respectively)		During		NR		NR

^a^The sample size of only the group exposed to the intervention.

^b^ED: emergency department.

^c^NR: not reported.

^d^HEADS-ED: Home, Education, Activities, Drugs, Suicidality, Emotions, and Discharge.

MMAT appraisal was conducted on 37 studies (abstracts for which no full text was available were excluded). Overall, the methodological quality of the studies varied: 30% (11/37) of the studies met ≤60% of the criteria outlined by the MMAT (lower methodological quality), and 70% (26/37) of the studies met >60% of the criteria (higher methodological quality) [26]. Reviewers’ ratings for each methodological quality criterion are presented in Multimedia Appendix 2 [28-33,35-39,41-46,49-51,54-66,68,73,74,76].

### Nature of Interventions

In all, 40% (22/55) of the EDCTs were designed for use after the ED visit when families were already at home. Over half of the tools targeted a single specific presenting complaint with asthma (15/55, 27%), fever (6/55, 11%), fractures (3/55, 6%), head injury (3/55, 6%), and otitis media (3/55, 6%), being the most frequently cited. In 13% (7/55) of studies, the discharge communication tool could be used for multiple presenting complaints (eg, patients with fever or head injury). Finally, 20% (11/55) of the tools were designed for use in any illness presentation. Some tools focused on a specific task or a narrow aspect of discharge communication (eg, medication regimen adherence) [70], whereas other tools were multi-focused with broader education, symptom monitoring, and care plan elements [57].

### Features and Technical Components of EDCTs

EDCTs support diverse communication pathways among providers, caregivers, patients, and other health care providers. Most of the tools targeted communication between an ED health care provider and the parent and caregiver (52/55, 94%) with a smaller number (6/55, 11%) also including communication with other health care providers (eg, family physician). One study of the Texas Emergency Department Asthma Surveillance programs [68] was an example of a multi-audience tool. In the study, the ED asthma educator used a Microsoft-based platform to individualize an education package for the caregiver (eg, select relevant video segments, figures and graphs, skills training, and motivational messaging). The plan was shared and discussed with the caregiver and then printed and sent to the family’s primary care provider. The educator could also generate and print a child-friendly version of the tailored written action plan for elementary-aged patients.

The primary technology modalities used were videos (20/55, 36%), kiosks (11/55, 20%), telephone calls (7/55, 13%), and text messaging (6/55, 11%). The remaining modalities include a wide range of offline stand-alone interactive computer programs and web platforms, mobile apps, interactive websites, and web-based games with multiple audiovisual elements. For example, a private multiplayer web-based social game called iCare Adventure uses noncompetitive gameplay for children and parents to explore therapeutic content on an iPad while in the ED waiting room [74].

A density map of presenting complaints targeted and primary technology modalities used to deliver the EDCT was generated (Tables S1 and S2 in Multimedia Appendix 3). Darker cells indicate where the largest number of studies have been conducted. Kiosks and videos are the 2 predominant modalities used as stand-alone asthma tools. Videos are the most studied modality for less frequently investigated medical concerns (eg, vomiting and pain).

There was substantial heterogeneity between the studies in the amount of time and effort required by patients and caregivers to use the tool. In all, 42% (23/55) of the tools required single-use, time-limited interaction (eg, watched one video once or entered information at a kiosk once). A total of 3 studies involved web platforms or interactive computer programs with larger educational components that allowed unlimited access (4/34, 12%). A program provided access over a specified follow-up period (eg, 120 hours after discharge) [43]. Multiple planned interactions with a tool typically involved a level of automation (eg, 2 automated text messages twice a week for 4 weeks) [51] or chronologically sequenced learning modules. All text messaging interventions were automated 1-way messaging of reminders with no option of bidirectional texting directly with a health care provider.

Duration of contact with the EDCT (ie, how long it took end users to complete expected tasks) was reported in 31% (17/55) of the studies. Among those that did report, the length of contact time for the patient and caregiver ranged from 110 seconds at a kiosk [37] to 80 minutes (where the latter measured the time to complete 5 web-based modules) [63]. A total of 44% (7/17) of those reporting took ≤5 minutes to complete, (4/17, 24%) took between 6 and 10 minutes, and 24% (4/17) took >10 minutes. The interventions (3/17, 18%) that took >12 minutes all specifically targeted asthma. Caregiver perceptions of frequency and duration were explored in a study of 243 families where 66 (27.2%) reported they had “had no time” to enter the website [64].

### Reported Impacts of EDCTs

There was significant heterogeneity in the reported purpose of deploying the EDCT and subsequent outcomes measured. Tables S1 and S2 in Multimedia Appendix 4 show a matrix of the outcomes measured per mode of EDCT technology delivered. The intensity of shading shows clusters (darker) versus gaps (lighter) within technologies.

The highest density of evidence was from the study of changes in caregiver knowledge after using video-based EDCTs (16 instances). The most assessed category of outcomes overall (including both primary and secondary) were caregiver and patient beliefs and attitudes (eg, confidence in managing at home and level of anxiety; 36 instances), knowledge and comprehension (eg, knowledge about symptoms; 29 instances), and health service use (eg, return visits to the ED; 25 instances). Health care provider satisfaction (5 instances) and cost (2 instances) were the least measured outcomes across all technology modalities.

Text message interventions were more likely to be measured on behavioral outcomes (eg, compliance with medication regime and follow-up appointment with primary care), whereas studies of video-based EDCTs typically used knowledge acquisition–related measures. A randomized controlled trial by Jové-Blanco et al [46] comparing video discharge instructions and standard verbal instructions for gastroenteritis showed that 49% of the intervention group and 18.6% of the control group answered all knowledge acquisition questions correctly (*P*<.001) [48]. However, EDCTs with greater technological sophistication do not always produce better knowledge outcomes. In a head-to-head trial of a static website and an interactive website about fever, Hart et al [65] unexpectedly found that both modalities had comparable knowledge gains, although caregivers were significantly more satisfied with the interactive version.

Measurement of knowledge outcomes occurred largely through bespoke self-report questionnaires that assessed general knowledge about symptoms, treatment options, medication and activity adherence, and service use [29]. Validated measures were most often cited in relation to patient health status (eg, Faces Pain Scale-Revised and Acute Otitis Media Severity of Symptom) and functioning (ie, Acute Asthma Behavioral Capability Questionnaire; Integrated Therapeutics Group Child Asthma Short Form). No adverse events were reported in any of these studies.

The directionality of primary outcomes pointed to positive effects for the primary measure (44/55, 80%) or no significant difference (10/55, 18%). Only one study reported negative findings with an increase in return visits to the ED after receiving the intervention compared with the control group (*P*<.03) [59]. Often, the authors reported positive primary outcomes but mixed results across secondary measures. For example, a study by Baker et al [39] showed increases in parental knowledge about fever but no significant differences in subsequent health service use. Similarly, a study by Zorc [56] showed significant changes in beliefs about the benefits of follow-up, but medication adherence and ED visits did not significantly differ at follow-up. Parental satisfaction with EDCTs was consistently moderate to high across all technology modalities. However, in some instances, respondents in the control condition, typically verbal or written discharge instructions, also reported high levels of satisfaction [46].

The ability to tailor information via the EDCT was particularly well received by parents when this option was available. For example, tailored mental health recommendations facilitated by electronic screening were perceived by parents as more useful (69.5% vs 30.5%) and more practical (71.8% vs 28.2%) compared with verbal instructions [34]. In another study, 23% of caregivers’ free text entries in the EDCT provided data that were not contained in the official electronic medical record [35].

Patient age [72], gender of caregiver [62], and parent education level [37] were the most frequently reported, statistically significant covariates vis-à-vis the primary outcome. Of note, only 3 studies reported collecting baseline data on the level of computer proficiency [28] and none in the past decade.

### Implementation Context Features Where EDCTs Have Been Used

In all, 42% (23/55) of the EDCTs were evaluated in at least one explicitly stated urban community. The majority were evaluated in pediatric EDs (37/55, 67%) or mixed ED settings (ie, both adult and pediatric populations, 13/55, 24%); the rest provided insufficient information to decide. English, Spanish, and Dutch were the only languages in which interventions were available and evaluated. No other culturally specific content or culturally adaptive features of the interventions were reported. The interventions (12/55, 22%) included baseline racial demographic factors, with most participants being African American or White. The EDCTs were most frequently delivered by research study staff (19/55, 35%), ED health care providers (15/55, 27%), or by computers or automated systems (8/55, 15%).

Very few interventions (3/55, 6%) were tested in studies that provided remuneration to participants. No studies have reported interoperability with other ICT systems within the ED or hospitals. The authors of 2 interventions (2/55, 4%) briefly mentioned sustainability planning, and 33% (18/55) stated that due consideration should be given to the technical performance of the system. Only 2 interventions (2/55, 4%) included details of direct costs; a study reported that per patient mean cost for videos was US $61 (SD US $36) versus US $31 (SD US $20) for phones; *P*<.001 [50]. Another study estimated the operating budget for the tool in “hundreds of dollars” [74]. Privacy and security were highlighted as necessary implementation context considerations in 11% (6/55) of the instances.

### Research, Practice, and Policy Implications Reported by Primary Authors

No direct policy or decision-making implications were explicitly discussed by the primary authors. High-level theming of future research directions posited by primary authors revealed three main directions: (1) more diverse sample populations that reflect a wider view of social determinants of health, (2) triangulation of data from sources outside of self-report (eg, primary care follow-up data and hospital administrative data), and (3) isolating the functionality of the tools to test the impact on engagement (eg, increase uptake). Practically, the authors generally endorsed the use of EDCTs, even if statistically significant findings were mixed or effect sizes were modest.

## Discussion

### Principal Findings

The primary aim of this review was to describe and assess evidence based on the EDCTs used in pediatric EDs. The evidence base included the principal features, measured outcomes, and implication contexts under which they were studied.

First, an important and promising finding of this review is that although the contextual complexity of EDs poses communicative challenges and risks, there is a growing body of evidence that EDCTS have been successfully integrated. Our review found at least five studies in each of the 4 major modality categories (ie, videos, kiosks, text messaging, and phone-based) and numerous presenting complaints that are among the most frequent reasons for ED visits reported in the literature (asthma, fever, head injury, fractures, pain, mental health, etc) [77]. In other words, there is growing breadth and depth of positive evidence.

The evidence base for newer technology modalities, kiosks, text messaging, and web-based games and apps is still maturing, with just under a third of all studies being conducted in the last 5 years. It is vital to monitor this evidence base as more automated and ambient technologies (eg, chat bots, wearables, and artificial intelligence) become normalized. Indeed, they are already being studied in ED communication for the adult population [78,79]. Our review adds to this dialogue by showing that technological sophistication may not necessarily result in clinically meaningful improvements. Videos and phone calls also produced positive changes. In fact, most EDCTs in this review reported at least some positive impact in 80% of cases and no adverse events. There is a need to move beyond demonstrating the known value of EDCTs and focus on *how* to optimize which tools for which populations, under which circumstances. This is supported by caregivers reporting high satisfaction regardless of modality or presenting concern. In other words, the technology modality used to support caregivers in discharge planning may be less crucial than the opportunity to engage with them.

Second, our review has shown that EDCTs have been largely assessed for changes in cognition (knowledge and beliefs), meaning that we know less about their impact on behavior (adherence to treatment regime), therapeutic relationship (caregiver-provider rapport), or service use. Our findings and overall methodological quality appraisal results point to the need for future meta-analyses to explore the magnitude and direction of effects within specific modalities. Such an analysis could support decision makers in determining which tools are fit for different primary purposes, reduction in nonurgent visits versus improved experiences of care. Caregivers may be highly satisfied with a tool and experience improved recall and comprehension, but this may not translate into fewer nonurgent visits to the ED in the future. The lack of description provided in primary studies related to implementation and environmental context features contributes to gaps in knowledge about the sustainability of these tools, particularly the costs associated with setup and ongoing operations.

Another significant finding of this review is that outcomes related to caregiver-provider rapport were understudied across all modalities and for all clinical presentations. This gap in the evidence is exacerbated by the few studies that assessed health care provider satisfaction with the tools in general. Assessment of their expectations and experiences with EDCTs may help illuminate barriers and enablers to uptake, as well as predictors of positive and negative client experiences. Recent work on quality pediatric communication in EDs [80] points to gaps in measures of care experiences in a complex, high-stress environment. Given the diverse implementation contexts for EDCTS found in this review, the development of quality standards for discharge communication should consider the role of electronic tools, which will undoubtedly continue to mediate and moderate care experiences in the future.

Finally, research designs for EDCTs need to incorporate mediators and moderators related to technological functions (eg, synchronicity, automation, visual aesthetics, and gamification) to determine the minimum viable functions. Our findings suggest that technological complexity is not necessarily better. Augmenting quantitative self-report survey data with observational, qualitative, and administrative data could help make sense of the aspects of these tools (ie, mechanisms of change) that drive the desired change. For example, there was some evidence that tools take >5 minutes for caregivers to complete (impact on workflow) and were administered by research team members rather than health care providers, giving us a slightly skewed view of real-world implementation. More work is needed to understand how the duration and frequency of interaction with tools (both provider and caregiver or patient) could be optimized for busy ED workflows without adding unnecessary complexity to the clinical pathways. Our review showed that over half of the EDCTs studied to date target a specific illness, but this could add burden to health care providers and caregivers who might then need to access and navigate a different tool for each presenting condition.

The findings of this review point to several high-impact future lines of research to address gaps, including (1) exploring how computer-mediated communication in pediatric emergency contexts impacts the quality dimensions of communication and rapport building (eg, sense of shared decision-making, empathy, and active listening), (2) meta-analysis of data subsets within a particular presenting illness field (eg, asthma) or within a single well-defined technology modality (eg, kiosks), (3) developing taxonomies for electronic discharge communication interventions that capture complex person-to-person and person-to-technology pathways, and (4) use of A or B (ie, split) testing to isolate specific technology features that may be driving outcomes so that the least intensive interventions necessary to achieve desired outcomes are pursued by developers and decision makers.

### Limitations

This study had several limitations. First, mapping the broad relevant literature parameters of *EDCTs* lacked clarity before the literature search. Terms related to technology, digital devices, and electronic communication were ambiguous in the literature, and our criteria were subject to significant revision during the initial search execution. This resulted in a less-focused initial title and abstract screening process. Second, the review included several study abstracts that were not published as full articles, limiting what data could be abstracted and fully analyzed. Finally, no taxonomies for presenting complaints have been validated or published in the literature; likewise, no taxonomies for electronic communication modalities are commonly used. Thus, our heat-map categorizations were based more on practical considerations and, to a lesser degree, on theoretically validated distinctions.

### Conclusions

To our knowledge, there has been no other systematic review of the broad evidence related to EDCTs in pediatric EDs. The findings demonstrate that a range of technologies are being used successfully. However, it is essential that trials of emerging technologies use robust and consistent measures of quality patient-provider communication, clinician experience, cost-effectiveness, and health service use so that influential evidence on these outcomes can accumulate.

## Appendix

Multimedia Appendix 1 MEDLINE @OVID search strategy.

Multimedia Appendix 2 Mixed Method Appraisal Tool quality appraisal profile.

Multimedia Appendix 3 Heat map of technology modalities and frequencies.

Multimedia Appendix 4 Heat map of intervention outcomes and frequencies.

## Abbreviations

ED: emergency department
EDCT: electronic discharge communication tool
ICT: information and communication technology
MMAT: Mixed Method Appraisal Tool
PRISMA: Preferred Reporting Items for Systematic Reviews and Meta-Analyses
