# Supplementary material for: Electronic Discharge Communication Tools Used in Pediatric Emergency Departments: Systematic Review
Source: JMIR Pediatr Parent. 2022 Jun 24;5(2):e36878. doi: 10.2196/36878 (PMC9270703; doi:10.2196/36878)
Supplement: Multimedia Appendix 1 [file pediatrics_v5i2e36878_app1.docx]

## Multimedia Appendix 1- Medline @OVID Search Strategy

| exp communication/ or blogging/ or communication barriers/ or digital divide/ or health communication/ or information dissemination/ or information literacy/ or health literacy/ or information seeking behavior/ or correspondence as topic/ or electronic mail/ or text messaging/ |
| --- |
| communications media/ or blogging/ or social media/ or audiovisual aids/ or telecommunications/ or electronic mail/ or telefacsimile/ or telemedicine/ or telephone/ or cell phone/ or text messaging/ or videoconferencing/ or webcasts as topic/ or wireless technology/ or exp data display/ |
| computer systems/ or computer communication networks/ or internet/ or internet access/ or social media/ |
| telephone/ or answering services/ or cell phone/ or text messaging/ |
| health communication/ or reminder systems/ |
| Hotlines/ |
| health education/ or consumer health information/ or health literacy/ or patient education as topic/ |
| hospital communication systems/ or exp hospital information systems/ |
| exp Medical Informatics Applications/ |
