# Supplementary material for: Electronic Discharge Communication Tools Used in Pediatric Emergency Departments: Systematic Review
Source: JMIR Pediatr Parent. 2022 Jun 24;5(2):e36878. doi: 10.2196/36878 (PMC9270703; doi:10.2196/36878)
Supplement: Multimedia Appendix 2 [file pediatrics_v5i2e36878_app2.docx]

## Multimedia Appendix 2 – MMAT Quality Appraisal Profile

|  | **Randomized**  **controlled-trials** | | | | | **Non-randomized** | | | | | **Quantitative**  **descriptive** | | | | | **Mixed**  **method** | | | | |
| --- | --- | --- | --- | --- | --- | --- | --- | --- | --- | --- | --- | --- | --- | --- | --- | --- | --- | --- | --- | --- |
| **Author, Yr** | **1** | **2** | **3** | **4** | **5** | **1** | **2** | **3** | **4** | **5** | **1** | **2** | **3** | **4** | **5** | **1** | **2** | **3** | **4** | **5** |
| Alqudah, 2014 | Y | Y | N | Y | C |  |  |  |  |  |  |  |  |  |  |  |  |  |  |  |
| Babcock, 2017 |  |  |  |  |  |  |  |  |  |  | Y | Y | Y | C | Y |  |  |  |  |  |
| Baker, 2009 | Y | N | C | C | Y |  |  |  |  |  |  |  |  |  |  |  |  |  |  |  |
| Bloch, 2013 | Y | Y | Y | C | Y |  |  |  |  |  |  |  |  |  |  |  |  |  |  |  |
| Boychuk, 2006 |  |  |  |  |  | Y | Y | Y | C | Y |  |  |  |  |  |  |  |  |  |  |
| Bucaro, 2014 |  |  |  |  |  |  |  |  |  |  | Y | Y | Y | Y | Y |  |  |  |  |  |
| Chande, 1994 | C | Y | Y | Y | Y |  |  |  |  |  |  |  |  |  |  |  |  |  |  |  |
| Fine, 2009 |  |  |  |  |  | Y | Y | Y | Y | Y |  |  |  |  |  |  |  |  |  |  |
| Golden-Plotnik 2018 | Y | C | Y | C | Y |  |  |  |  |  |  |  |  |  |  |  |  |  |  |  |
| Goldman, 2005 |  |  |  |  |  |  |  |  |  |  | Y | Y | Y | Y | Y |  |  |  |  |  |
| Goldman, 2014 | Y | Y | Y | C | Y |  |  |  |  |  |  |  |  |  |  |  |  |  |  |  |
| Hart, 2019 | Y | Y | Y | Y | Y |  |  |  |  |  |  |  |  |  |  |  |  |  |  |  |
| Hoek ,2020 |  |  |  |  |  | Y | Y | Y | Y | N |  |  |  |  |  |  |  |  |  |  |
| Ismail, 2016 | Y | C | Y | Y | Y |  |  |  |  |  |  |  |  |  |  |  |  |  |  |  |
| Jones, 1989 | C | C | Y | Y | Y |  |  |  |  |  |  |  |  |  |  |  |  |  |  |  |
| Joshi, 2009 |  |  |  |  |  |  |  |  |  |  | Y | Y | Y | C | Y |  |  |  |  |  |
| Jove-Blanco, 2021 | Y | Y | Y | C | Y |  |  |  |  |  |  |  |  |  |  |  |  |  |  |  |
| Kearns, 2021 |  |  |  |  |  |  |  |  |  |  | C | Y | Y | Y | Y |  |  |  |  |  |
| Khan, 2004 | Y | Y | Y | Y | Y |  |  |  |  |  |  |  |  |  |  |  |  |  |  |  |
| Kwok, 2018 |  |  |  |  |  |  |  |  |  |  | C | N | Y | C | Y |  |  |  |  |  |
| Lawrence, 2009 |  |  |  |  |  | Y | Y | Y | Y | C |  |  |  |  |  |  |  |  |  |  |
| Lion, 2015 | Y | N | Y | Y | Y |  |  |  |  |  |  |  |  |  |  |  |  |  |  |  |
| Lund, 2013 | Y | C | N | N | Y |  |  |  |  |  |  |  |  |  |  |  |  |  |  |  |
| Macy, 2011 | C | Y | Y | Y | Y |  |  |  |  |  |  |  |  |  |  |  |  |  |  |  |
| Morrison, 2021 |  |  |  |  |  | Y | Y | C | C | Y |  |  |  |  |  |  |  |  |  |  |
| Mortenson, 2016 | Y | Y | Y | Y | Y |  |  |  |  |  |  |  |  |  |  |  |  |  |  |  |
| Porter, 2004 |  |  |  |  |  |  |  |  |  |  |  |  |  |  |  | Y | Y | Y | Y | Y |
| Porter, 2006 |  |  |  |  |  | Y | Y | Y | Y | Y |  |  |  |  |  |  |  |  |  |  |
| Porter, 2008 |  |  |  |  |  | Y | Y | Y | Y | Y |  |  |  |  |  |  |  |  |  |  |
| Sinha, 2014 | Y | Y | Y | N | Y |  |  |  |  |  |  |  |  |  |  |  |  |  |  |  |
| Sockrider, 2006 | Y | Y | Y | C | C |  |  |  |  |  |  |  |  |  |  |  |  |  |  |  |
| Taylor, 2015 |  |  |  |  |  | Y | Y | Y | Y | Y |  |  |  |  |  |  |  |  |  |  |
| Wolff, 2016 | Y | Y | Y | N | Y |  |  |  |  |  |  |  |  |  |  |  |  |  |  |  |
| Wong, 2004 | Y | Y | Y | C | Y |  |  |  |  |  |  |  |  |  |  |  |  |  |  |  |
| Wood, 2017 |  |  |  |  |  | Y | Y | C | C | Y |  |  |  |  |  |  |  |  |  |  |
| Wood,2020 |  |  |  |  |  | Y | Y | C | C | Y |  |  |  |  |  |  |  |  |  |  |
| Zorc, 2009 | Y | Y | N | N | Y |  |  |  |  |  |  |  |  |  |  |  |  |  |  |  |

Y= that the criterion is met (yes) , N= the criterion is not met (no), C= there is not enough information in the paper to judge if the criterion is met or not (Can’t tell).
