# Supplementary material for: Electronic Discharge Communication Tools Used in Pediatric Emergency Departments: Systematic Review
Source: JMIR Pediatr Parent. 2022 Jun 24;5(2):e36878. doi: 10.2196/36878 (PMC9270703; doi:10.2196/36878)
Supplement: Multimedia Appendix 3 [file pediatrics_v5i2e36878_app3.docx]

**Table S1.** ED visit presenting concern by primary technology modality.

| Condition targeted | Kiosk | Video | Phone | Web | Computer | Text | Other^a^ |
| --- | --- | --- | --- | --- | --- | --- | --- |
| Head injury |  |  |  |  |  |  |  |
| Asthma |  |  |  |  |  |  |  |
| Otitis media |  |  |  |  |  |  |  |
| Fever |  |  |  |  |  |  |  |
| Fracture |  |  |  |  |  |  |  |
| Multiple^b^ |  |  |  |  |  |  |  |
| Nonspecific^c^ |  |  |  |  |  |  |  |
| Other^d^ |  |  |  |  |  |  |  |

^a^ Other includes photodocumentation, games and other types of technologies

^b^Cases where the same tool could be used for more than one condition and those conditions were listed.

^c^Cases where the tool could be used for any condition.

^d^Cases where the tool was used for a specific condition not captured under head injury, asthma, otitis media, fever, or fracture.

**Table S2.** ED visit presenting concern by primary technology modality frequencies

| Condition Targeted | Kiosk | Video | Phone | Web | Comp. | Text | Other |
| --- | --- | --- | --- | --- | --- | --- | --- |
| Head Injury | 1 | 1 | 0 | 1 | 0 | 0 | 0 |
| Asthma | 5 | 5 | 1 | 0 | 1 | 2 | 1 |
| Otitis Media | 0 | 1 | 2 | 0 | 0 | 0 | 0 |
| Fever | 0 | 2 | 0 | 1 | 3 | 0 | 0 |
| Fracture | 0 | 1 | 0 | 0 | 1 | 1 | 0 |
| Multiple | 2 | 3 | 2 | 0 | 0 | 0 | 0 |
| Non-Specific | 2 | 3 | 2 | 1 | 0 | 2 | 1 |
| Other | 1 | 4 | 0 | 0 | 0 | 1 | 1 |
