# Supplementary material for: Electronic Discharge Communication Tools Used in Pediatric Emergency Departments: Systematic Review
Source: JMIR Pediatr Parent. 2022 Jun 24;5(2):e36878. doi: 10.2196/36878 (PMC9270703; doi:10.2196/36878)
Supplement: Multimedia Appendix 4 [file pediatrics_v5i2e36878_app4.docx]

**Table S1.** Outcomes measured by technology modality.

| Outcome measure categories | Kiosk | Video | Phone | Web | Computer | Text | Other^a^ |
| --- | --- | --- | --- | --- | --- | --- | --- |
| Caregiver or/patient behavior |  |  |  |  |  |  |  |
| Caregiver or patient beliefs and attitudes |  |  |  |  |  |  |  |
| Caregiver or patient satisfaction |  |  |  |  |  |  |  |
| Caregiver or patient knowledge |  |  |  |  |  |  |  |
| Child’s health status |  |  |  |  |  |  |  |
| Rapport with provider |  |  |  |  |  |  |  |
| Provider satisfaction |  |  |  |  |  |  |  |
| Cost |  |  |  |  |  |  |  |
| Health Service use |  |  |  |  |  |  |  |

^a^ Includes games, mobile app, and electronic medical records.

**Table S2**. Outcomes measured by technology modality frequencies

| Outcomes | Kiosk | Video | Phone | Web | Comp. | Text | Other |
| --- | --- | --- | --- | --- | --- | --- | --- |
| Caregiver/Patient Behaviour | 1 | 4 | 3 | 0 | 1 | 4 | 3 |
| Caregiver/patient Beliefs an Attitudes | 5 | 14 | 7 | 4 | 5 | 1 | 4 |
| Caregiver/patient Satisfaction | 3 | 10 | 2 | 3 | 1 | 0 | 3 |
| Caregiver/patient Knowledge & Comprehension | 1 | 16 | 3 | 2 | 5 | 0 | 2 |
| Rapport with Provider | 4 | 5 | 1 | 0 | 0 | 0 | 1 |
| Provider Satisfaction | 1 | 1 | 1 | 0 | 0 | 0 | 1 |
| Cost | 0 | 1 | 0 | 0 | 0 | 0 | 0 |
| Health Service Utilization | 2 | 7 | 5 | 2 | 2 | 3 | 4 |
